# When wet meets dry: An Ivuna-like impactor triggered volatile loss on the angrite parent body

**DOI:** 10.1126/sciadv.aeb1432

**Published:** 2026-05-29

**Authors:** Ke Zhu (朱柯), Qi Chen, Tetsuya Yokoyama, Akira Yamaguchi, Audrey Bouvier, Lu Chen

**Affiliations:** ^1^ State Key Laboratory of Geological Processes and Mineral Resources, Hubei Key Laboratory of Planetary Geology and Deep-Space Exploration, School of Earth and Planetary Sciences, China University of Geosciences, Wuhan 430074, China.; ^2^ School of Earth, Atmosphere and Environment, Monash University, Melbourne, VIC 3800, Australia.; ^3^ School of Earth Sciences, University of Bristol, Wills Memorial Building, Queen’s Road, Bristol BS8 1RJ, UK.; ^4^ Center for Advanced Radiation Sources, The University of Chicago, Chicago, IL, USA.; ^5^ Department of Earth and Planetary Sciences, Institute of Science Tokyo, Tokyo 152-8551, Japan.; ^6^ National Institute of Polar Research, Tachikawa, Tokyo 190-8518, Japan.; ^7^ Bayerisches Geoinstitut, University of Bayreuth, Universitatsstrasse 30, Bayreuth 95447, Germany.; ^8^ Wuhan SampleSolution Analytical Technology Co. Ltd., Wuhan, China.

## Abstract

Angrite meteorites remain the “driest” known planetary materials in the Solar System. Recent mineralogical and oxygen (O) isotope data suggest that the angrite parent body (APB) experienced a major impact, raising questions about the identity of the impactor. We report high-precision mass-independent chromium (ε^54^Cr) isotope data for eight angrites and their components. Samples from deeper APB regions exhibit uniform ε^54^Cr values (−0.40 ± 0.03), representing the mantle composition, whereas matrix materials from quenched angrites show anomalous ε^54^Cr values (0.27 ± 0.06 and −0.15 ± 0.08), likely reflecting impactor contamination. Combined Cr-O isotope data point to a CI (Ivuna-type) chondritic impactor—the “wettest” known Solar System material. Although such an impact could introduce volatiles, high-energy conditions likely caused extensive volatile loss, preserving the angrites’ dry nature. CI contamination may also make quenched angrites unsuitable time anchors for siderophile short-lived chronometers, whereas the other “contamination-free” angrites yield a precise age of 4561.0 ± 0.9 million years, dating the formation of their mantle source.

## Teaser

Chromium isotopes in angrites reveal impact and mixing between the angrite parent body and the CI chondrite material.

## INTRODUCTION

Planetesimals formed and differentiated at the beginning of the Solar System, dating back to ∼4.57 billion years ago (*1*–*7*), recording important information about early Solar System evolution. Recently, the samples from Ryugu and Bennu (*8*, *9*) provide direct samples from these primitive chondritic asteroids. The analysis of samples from differentiated (melted) asteroids still mainly relies on meteorites. Among achondrite meteorites, angrites are a relatively small group of meteorites that are believed to originate from a differentiated parent body. The angrite parent body (APB) underwent core formation around 2 million years after the Solar System’s formation (*10*), with mantle differentiation occurring at 4563.3 ± 0.4 million years ago (Ma) dated by bulk ^53^Mn-^53^Cr isochron, about 4 to 5 million years after Solar System formation (*11*), and magmatism continuing until ∼4556 Ma (*3*, *10*, *12*–*14*). All angrites are characterized by the highest Fe/Mn and lowest K/U and Rb/Sr ratios among achondrites, indicating their nature of highly moderately volatile element depleted (*15*, *16*). The homogeneous, mass-independent isotopic compositions of O, Cr, and Ti in bulk angrites suggest a magma ocean phase on their parent body (*11*, *17*–*19*). Because of their homogenized isotopic characteristics, rapid cooling and crystallization, and precise absolute ages, quenched angrites are widely used as time anchors for short-lived chronometers such as the ^26^Al-^26^Mg (*T*_1/2_ = 0.7 million years) and ^53^Mn-^53^Cr (*T*_1/2_ = 3.7 million years) systems (*2*, *4*, *13*, *20*, *21*).

Angrites are typically classified into plutonic and quenched types, with plutonic angrites displaying coarse grained, near-equilibrium textures, and slower cooling rates compared to quenched angrites (*22*). In recent years, with observation of more angrite meteorites, additional petrological types have been identified, including intermediate [e.g., Northwest Africa (NWA) 10463], dunitic (i.e., NWA 8535), diabasic (e.g., NWA 12320), and subgroups in quenched (high-Mg and low-Mg) and plutonic (Angra dos Reis and others) angrites (*22*–*25*). In detail, the quenched angrites usually exhibit two phases: mega-olivine (up to centimeter-sized) and matrix (*26*). Rider-Stokes *et al.* (*27*). separated the matrix and mega-olivine from quenched angrites and conducted high-precision mass-independent oxygen isotope analyses. They found that the matrix has a Δ^17^O value of −0.003 ± 0.020 per mil (‰), systematically higher than the average Δ^17^O values of plutonic angrites and mega-olivine (−0.066 ± 0.016‰). Combined with the recrystallization textures with low level of shock reworking observed in the matrix, Rider-Stokes *et al.* (*27*) proposed that the APB experienced a major planetary collision. During this event, an impactor with a more positive Δ^17^O composition “contaminated” the surface matrix material, altering its Δ^17^O signature. This impact may have played a crucial role in the differentiation and volatile depletion of the APB. The potential contamination from the impactor could also affect the validity of quenched angrites as reliable “time anchors” (*28*, *29*), due to the addition of exotic material. However, the specific nature and origin of the impactor remain unknown.

**Fig. 1.**
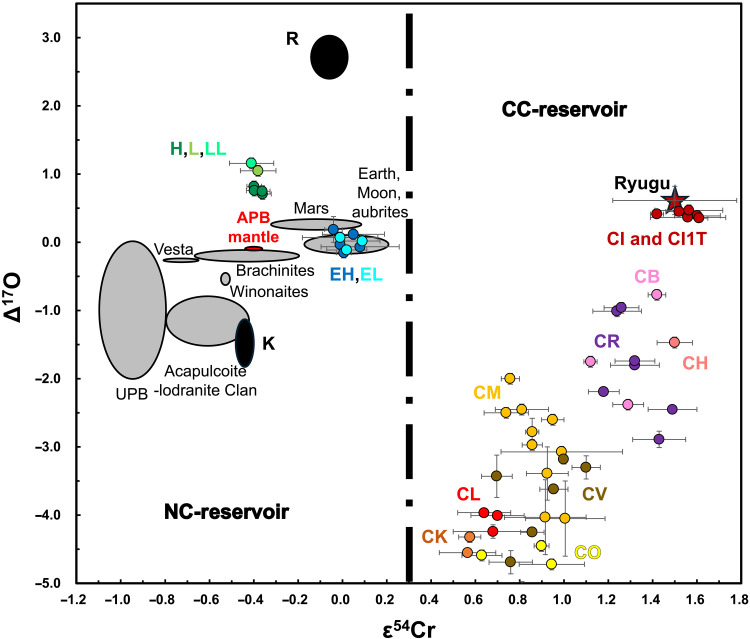
ε^54^Cr and ∆^17^O compositions of the Solar System materials, including chondrites (filled circles), as well as achondrites, terrestrial, and lunar samples (gray ellipses, except for the red ellipse representing the APB mantle). The mantle of the APB is marked in red. In this figure, we only used peer-reviewed published data. The data sources can be found in the literature [(*8*, *31*, *33*, *35*, *36*, *46*, *79*, *110*–*113*) and references therein]. CI1T chondrites may be thermally metamorphosed CI1 chondrites from an impact (*33*, *79*). For Cr isotope data of Ryugu (red star), we only take the samples with mass more than 10 mg, due to the potential sample heterogeneities. Abbreviations: CC, carbonaceous chondrite-like; NC, non–carbonaceous chondrite-like; APB, angrite parent body; UPB, ureilite parent body; R, Rumuruti chondrites; K, Kakangari chondrites. The dashed line represents a boundary of carbonaceous (outer Solar System) and noncarbonaceous (inner Solar System) bodies.

Mass-independent Cr isotope measurements may be a robust method for tracing the APB’s impactor. Solar System bodies exhibit a wide range of mass-independent ^54^Cr/^52^Cr ratios relative to terrestrial standard: NIST SRM 979 (expressed as ε^54^Cr; per ten thousand isotope deviations), with values ranging from 0.4 to 1.6 for carbonaceous chondrite-like (CC) material and Ryugu; ∼0 for the Earth-Moon system, main-group aubrites, Rumuruti, and enstatite chondrites; and down to −1.1 for ordinary, Kakangari chondrites, and noncarbonaceous (NC) differentiated bodies (*5*, *8*, *11*, *30*–*47*). For example, ε^54^Cr measurements show that CC material was added to enstatite chondrule precursors (*48*). As a siderophile (at high pressure) and compatible element, Cr is depleted in planetary crusts relative to chondrites, making Cr isotopes particularly sensitive to tracing chondritic additions to surfaces of differentiated bodies (*49*). For instance, ε^54^Cr measurements indicate that CC material contaminated the crusts of early Earth (*50*–*53*) and Mars (*35*). Because Δ^17^O and ε^54^Cr signatures vary among Solar System materials (Fig. 1), the combination of both is effective for identifying the APB’s impactor.

In this study, we separated mega-olivine and matrix minerals from quenched angrites and measured their mass-independent Cr isotope compositions. We also analyzed plutonic and dunitic angrites to compare ε^54^Cr compositions between mantle-derived and potentially altered crustal materials. These data provide constraints on the impactor’s origin and its influence on APB evolution. Furthermore, ε^53^Cr data from a more diverse set of angrites can help refine the Mn-Cr bulk isochron (*11*).

## RESULTS

**Fig. 2.**
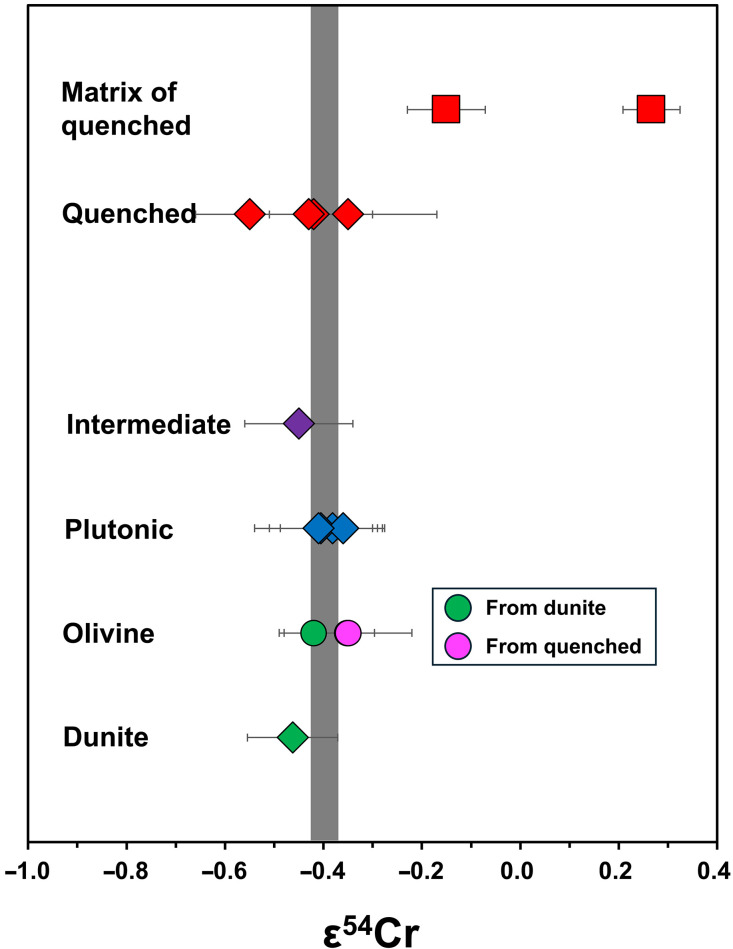
ε^54^Cr signature of different types of angrite samples. The gray bar indicates the mantle composition of APB (−0.40 ± 0.03, 2SE), averaged by the ε^54^Cr values of samples excluding bulk quenched angrites and matrix samples of quenched angrites.

**Fig. 3.**
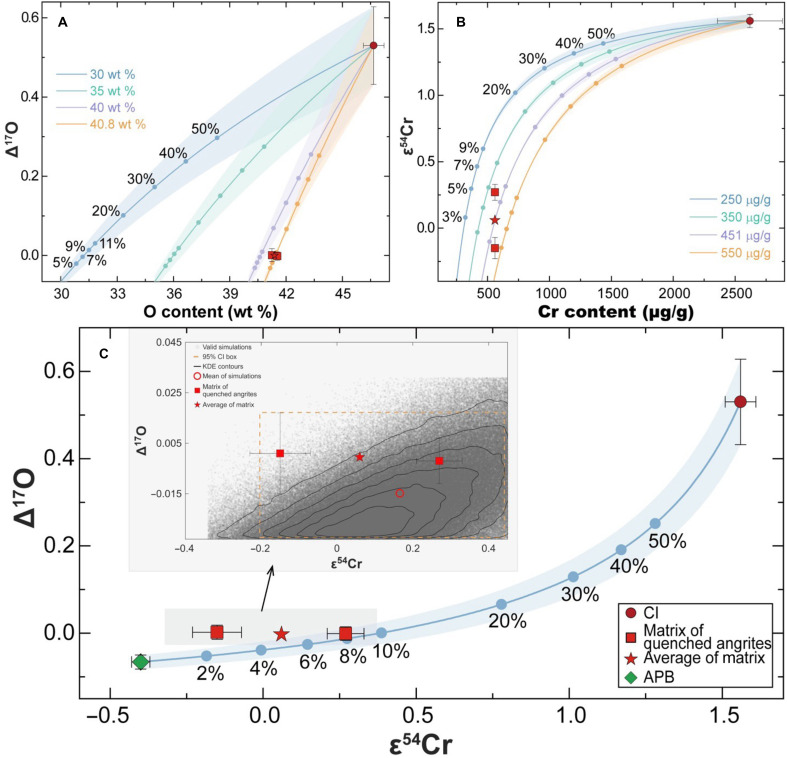
Binary mixing models based on Monte Carlo simulations for Δ^17^O and ε^54^Cr isotopic systems using the APB mantle (green diamond) and CI (red circle) as two endmembers. Red squares: the two matrices of quenched angrites (A-881371 and A-12209); red star: their mean (used as the matrix constraint in the inversion). (**A**) Δ^17^O versus O content (wt %), where O is calculated stoichiometrically from ICP-MS major/minor element abundances (table S1) and Δ^17^O for the APB and matrix is taken from Rider-Stokes *et al.* (*27*). (**B**) ε^54^Cr versus Cr content (μg/g) across a range of assumed APB O and Cr concentrations. Numbers along curves denote the CI fraction (%). Single-system fits give *f*_CI_ = 9.54 ± 3.61% and 5.01 ± 2.49% for O and Cr isotopic systems, respectively. (**C**) Joint Monte Carlo inversion. The blue curve and shaded band show the forward mixing model and its uncertainty. Gray points represent valid simulations that simultaneously match the observed matrix-mean value within 2σ in both isotopic systems. Kernel Density Estimation (KDE) contours in the inset show the solution density [orange dashed box; 95% confidence interval (CI)]. The joint model yields a best-fit *f*_CI_ of 6.29 ± 1.97%, consistent but more tightly constrained than the single-isotope models (see the Supplementary Materials for details).

All Cr isotope data are reported in Table 1, and the elemental data are listed in table S1. The mega-olivines from Asuka (A) 881371, A 12209 (quenched), and one olivine grain from NWA 8535 (dunite) have ε^54^Cr values of −0.42 ± 0.07, −0.35 ± 0.13, and −0.35 ± 0.05, respectively. These values overlap with those of bulk NWA 8535 (ε^54^Cr = −0.46 ± 0.09), as well as other plutonic and intermediate angrites analyzed in this study and in the literature (*11*, *36*). All angrite samples exhibit homogeneous ε^54^Cr values, averaging −0.40 ± 0.08 (2SD) ± 0.03 (2SE, *N* = 9). The matrix samples from A 881371 and A 12209 show elevated ε^54^Cr values of 0.27 ± 0.06 and −0.15 ± 0.08, respectively, which are resolvably higher than those of the mega-olivines and other angrites (Figs. 2 and 3).

**Table 1.** Mn-Cr data of angrite samples and data quality control standards. Note that Erg Chech 002 is powdered from >1 g of chip. The Cr isotope composition of MIL 07001 is measured only once, so we use the 2SD uncertainty of NIST SRM 979 in the measurement sessions for the errors of its Cr isotope data. Sample names not labeled with “Olivine” or “Matrix” refer to bulk samples. The O isotope composition of the APB mantle is estimated as −0.066 ± 0.016‰, and all the O isotope data are from (*27*). The nonzero ε^53^Cr and ε^54^Cr values of terrestrial standard rocks (i.e., DTS-1 and JP-1) can be caused by equilibrium Cr isotope fractionation during the preparation of the NIST SRM 979 standard (*31*, *108*). The mass-independent Cr isotope composition of bulk silicate Earth is estimated as ε^53^Cr = 0.04 ± 0.08 and ε^54^Cr = 0.09 ± 0.12, 2SD (*4*, *31*, *36*, *37*).

Sample	Petrology	Mass (mg)	Mg#	Cr content (μg/g)	Fe/Cr	^55^Mn/^52^Cr	ε^53^Cr	2SE	ε^54^Cr	2SE	*N*/References	∆^17^O (‰)	2SD
NWA 8535	Dunite	28.7	0.83	5673	17.8	0.24	**0.01**	0.03	**−0.46**	0.09	4	−0.070	0.011
Olivine-NWA 8535	Olivine	10.9	0.86	3060	30.3	0.41	**0.04**	0.07	**−0.35**	0.05	4		
Matrix-A 881371	Quenched	34.0	0.42	557	279.3	3.66	**1.19**	0.03	**0.27**	0.06	5	−0.002	0.009
Olivine-A881371	Olivine	15.0	0.88	1544	53.4	0.74	**0.23**	0.04	**−0.42**	0.07	4	−0.067	0.026
Matrix-A 12209	Quenched	35.8	0.39	566	283.9	3.81	**1.06**	0.02	**−0.15**	0.08	4	0.001	0.016
Olivine-A 12209	Olivine	7.2	0.89	1560	50.6	0.72	**0.26**	0.02	**−0.35**	0.13	3	−0.068	0.014
NWA 14758	Plutonic	53.7	0.49	2237	105.0	0.91	**0.14**	0.05	**−0.38**	0.11	4		
Rafsa 005	Plutonic	33.7	0.53	1732	125.8	1.26	**0.18**	0.03	**−0.41**	0.10	5		
Erg Chech 002	Ungrouped Achondrite	∼50	0.53	3526	21.4	1.08	**0.31**	0.03	**−0.79**	0.03	6		
MIL 07001	Diogenite	33.1					**0.09**	0.06	**−0.58**	0.12	1		
DTS-1	Terrestrial	∼50					**0.07**	0.03	**0.21**	0.09	4		
JP-1	Terrestrial						**0.05**	0.02	**0.13**	0.04	4/Zhu *et al.* (*33*)		
Allende	CV chondrite	73.2					**0.12**	0.03	**0.91**	0.05	3/Zhu *et al.* (*33*)		

**Fig. 4.**
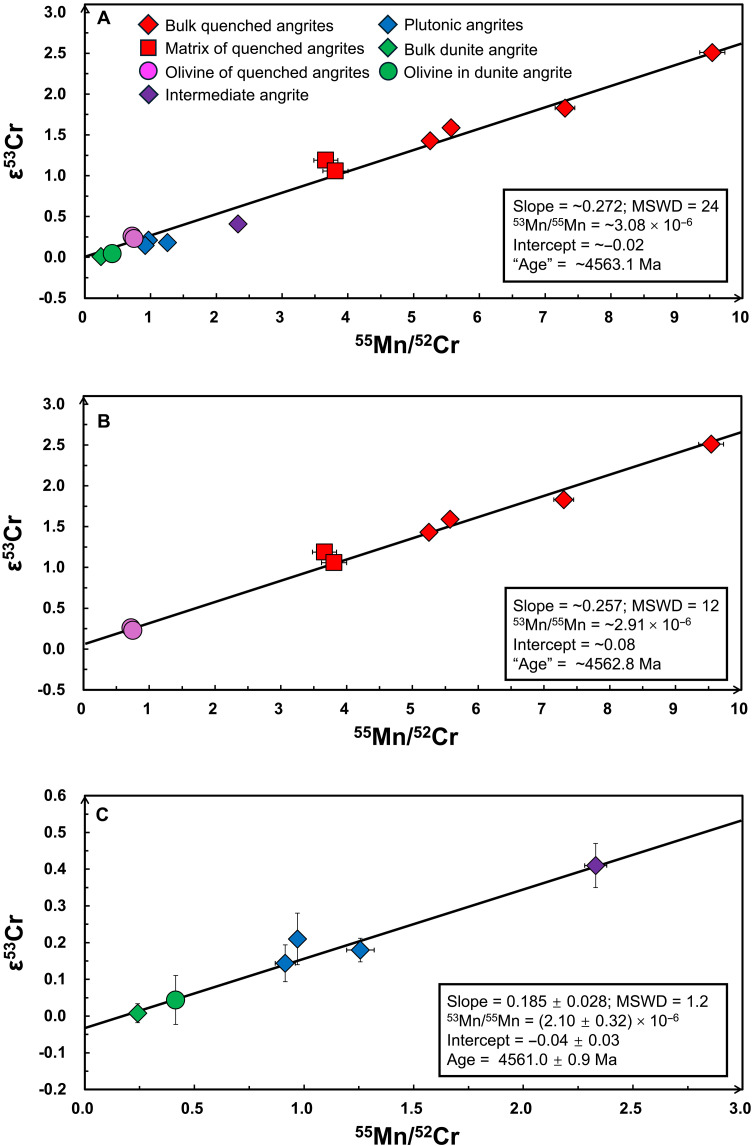
^53^Mn-^53^Cr correlation lines and isochrons of angrites and their components. The symbols are the same in Fig. 2. (**A**) All angrites and their components. (**B**) Matrix and mega-olivines in quenched angrites and bulk. (**C**) Bulk dunite, olivine in dunite, and plutonic and intermediate angrites. The “age” in (A) and (B) is translated by the regressed slope of the correlation lines, and because of the potential mixing lines and contamination of CI-like material, we do not anticipate their time significance.

Bulk NWA 8535 has the lowest ^55^Mn/^52^Cr ratio (0.24) and ε^53^Cr value (0.01 ± 0.05) among all angrite samples, closely matching its olivine grain, which has a ^55^Mn/^52^Cr ratio of 0.41 and an ε^53^Cr value of 0.04 ± 0.07. Mega-olivines in A 881371 and A 12209 have lower Cr contents (∼1500 μg/g) and higher ^55^Mn/^52^Cr ratios (∼0.7) than the olivine in NWA 8535 (3060 μg/g Cr). All angrite samples, including literature data, show a broad correlation between ^55^Mn/^52^Cr ratios and ε^53^Cr values [mean square weighted deviation (MSWD) = 24; Fig. 4A]. However, when considering only the olivine samples from NWA 8535, and the plutonic and intermediate angrites, they define a well-resolved ^55^Mn/^52^Cr-ε^53^Cr correlation line with a slope of 0.185 ± 0.028 and a *y* intercept of −0.04 ± 0.03 (MSWD = 1.2).

## DISCUSSION

### The ε^54^Cr composition of the APB mantle

Before discussing the importance of the Cr isotope data from angrite samples, we first confirm that their Cr isotope compositions are primitive and not altered by cosmogenic effects (*54*). Cosmogenic alteration of Cr isotopes occurs via two mechanisms: spallation (*40*, *55*) and neutron capture (*37*). These processes can be monitored using the Fe/Cr ratio and cosmic ray exposure age (CREA) and Sm isotopes, respectively. We plotted Fe/Cr ratio × CREA versus ε^54^Cr values for the angrite samples (fig. S1). The CREA data of angrites come from (*56*) and references therein. Except for the matrix of A 881371, all other samples, including those with higher Fe/Cr ratios and longer CREAs, exhibit homogeneous ε^54^Cr values (*11*). In addition, previous studies showed that three angrites—NWA 4590, NWA 4801, and D’Orbigny—have homogeneous and unmodified Sm isotope ratios (*57*), further supporting that cosmogenic effects have not substantially altered the Cr isotopic compositions of these samples. We also plot the ε^53^Cr and ε^54^Cr data of angrites (fig. S2), together with literature values (*11*). The absence of correlations between ε^53^Cr and ε^54^Cr indicates that the anomalously high ε^54^Cr values in the matrices of A 881371 and A 12209 are unlikely to result from artifacts introduced during column chemistry (*58*) or thermal ionization mass spectrometry (TIMS) analyses (*31*, *59*, *60*). For the other bulk quenched angrites, their Cr isotope compositions are expected to be dominated by their Cr-rich mega-olivines ([Cr] of ∼1500 μg/g, relative to ∼500 μg/g in matrix; table S1). Because these olivines are mantle derived and free from contamination, they should largely control the bulk Cr isotope signatures.

Because the matrix of quenched angrites may have been modified by an impactor (*27*), potentially including Cr (see discussion below), we reevaluate the ε^54^Cr mantle composition of the APB by excluding the bulk quenched angrites (*11*). We selected all deep-origin olivine samples from quenched angrites and a dunite, along with the bulk dunite, intermediate, and plutonic angrites. These samples yield an average ε^54^Cr value of −0.40 ± 0.03 (2SE, *N* = 9; Figs. 1 and 2). This revised estimate provides a more accurate and precise ε^54^Cr mantle composition for the APB compared to a previous study (*11*).

### CI-like impactor contaminated APB’s crust

The anomalous ε^54^Cr values observed in the matrix of quenched angrites are consistent with the anomalous Δ^17^O values measured in the matrix of three quenched angrites—A 881371, A 12209, and NWA 12320 (*27*)—which have been interpreted as the result of mixing with an impactor having a distinct Δ^17^O composition. Considering only the Δ^17^O systematics, the impactor must have had higher Δ^17^O values than the APB, which can be both inner and outer Solar System materials such as ordinary chondrites, Rumuruti chondrites, Mars, CI chondrites, and some CI1T chondrites (previously CY1) (*33*, *61*–*63*). Although Earth, the Moon, enstatite chondrites, and aubrites have similar Δ^17^O values (∼0) to the matrix of quenched angrites (*64*, *65*), they are unlikely to be the impactor source as the mixing component cannot be entirely composed of impactor material. Notably, some carbonaceous achondrites with CI-like ε^54^Cr values (1.3 to 1.5), such as NWA 2976/6693 (*44*), NWA 011 (*66*, *67*), and Tafassassetites (*42*, *47*, *68*), can also be excluded due to their low Δ^17^O values (<0), which do not fit the observed mixing trend.

Considering both Δ^17^O and ε^54^Cr systematics, the impactor is best represented by CI-like material, including CI1T chondrites and Ryugu-like material (Figs. 1 and 3). Angrites and CI chondrites formed in the inner and outer Solar System, respectively (*11*, *19*, *36*). Their impact suggests that early material was transferred from the outer to the inner Solar System or that their parent planetesimals collided in the asteroid belt (*69*). Although both matrix samples of quenched angrites have elevated ε^54^Cr values compared to the APB mantle, they do not overlap: The difference of ∼0.4 ε-units (∼40 μg/g) suggests a heterogeneous and inefficient mixing process. This interpretation is supported by large differences in highly siderophile element abundances (HSE; which is very high in chondrites) among quenched angrites, e.g., NWA 1296 has substantially lower HSE contents than some other quenched angrites (*70*). The heterogeneous ε^54^Cr and HSE signatures suggest rapid cooling of the melt following impact, which would inhibit homogenization of isotopic and elemental signals associated with metals. In contrast, the matrix samples exhibit homogeneous Δ^17^O values at the level of ∼20 μg/g, possibly due to the faster diffusion rate of oxygen compared to metals like Cr. However, according to the HSE data of quenched angrites (*70*), quantitatively, CI-like material addition should only range from ∼0.01% (e.g., A 881371) to ∼1% (e.g., D’Orbigny). The estimates of CI material addition obtained from HSE budgets are systematically lower than those obtained from the Δ^17^O-ε^54^Cr systematics (∼5.8%). This suggests that some HSE added from CI material might have partitioned into the core of APB during the mantle remelting and magma ocean (*11*, *17*, *18*) after impact.

To further evaluate the mixing process between the original APB (before impact) and CI-like material, we quantitatively modeled binary mixing in Δ^17^O-ε^54^Cr space. The primitive APB has well-established values of Δ^17^O = −0.066 ± 0.016‰ (*27*) and ε^54^Cr = −0.40 ± 0.03, whereas CI chondrites have Δ^17^O = 0.530 ± 0.098‰ (*62*) and ε^54^Cr = 1.56 ± 0.05 (*31*, *36*). CI chondrites also have average O and Cr contents of 46.66 ± 0.55 wt % (*71*) and 2620 ± 262 μg/g (*72*), respectively. The O content of the matrix in A 881371 and A 12209 was calculated to be 41.40 ± 0.21 wt % based on inductively coupled plasma mass spectrometry (ICP-MS) data in this study (table S1), assuming all major and minor elements are bound in simple oxides (e.g., FeO, Al_2_O_3_, and MnO).

To evaluate binary mixing between the isotopic endmembers, we applied a forward Monte Carlo inversion approach to both individual and combined isotope systems. Isotopic ratios were calculated as concentration-weighted mixtures, following the generalized two-component mixing framework of (*73*). Modeling of Δ^17^O alone, incorporating O concentrations and uncertainties, yielded a CI contribution fraction (*f*_CI_) of ∼9.54 ± 3.61% (Fig. 3A), whereas the independent inversion based on ε^54^Cr and Cr concentrations gives a *f*_CI_ of 5.01 ± 2.49% (Fig. 3B). These estimates overlap within their 2σ uncertainties, indicating that both isotope systems are compatible with a common two-component CI-APB mixing scenario. However, their central values differ, likely reflecting differing sensitivities of the O and Cr systems to small contributions from the isotopically distinct endmember.

To improve precision, we developed a joint Monte Carlo inversion framework incorporating both ε^54^Cr and Δ^17^O, along with their respective elemental concentrations (see the Supplementary Materials). This combined model reduces ambiguity by enforcing agreement between both isotopic systems, yielding a refined *f*_CI_ estimate of 6.29 ± 1.97% (Fig. 3C), with physically consistent endmember concentrations. The agreement between observed and modeled isotopic values within 2σ uncertainty, along with convergence across both systems, supports a robust binary mixing interpretation. Full methodological details, including uncertainty propagation and inversion statistics, are provided in the Supplementary Materials (figs. S3 to S5).

On the basis of the isotopic dichotomy observed among Solar System materials (*36*, *74*, *75*), the parent body of CI chondrites is inferred to have accreted in the outer Solar System. The presence of CI-like material mixed into the early-differentiated APB, which formed in the inner Solar System, therefore implies efficient and large-scale mass transport from the outer to the inner Solar System at very early times. This interpretation is consistent with the positive ε^54^Cr values observed in some chondrules from enstatite chondrites (*48*). Conversely, the occurrence of Ca-Al–rich inclusions in carbonaceous chondrites (*76*), together with isotopic evidence from chondrules (*77*, *78*), indicates transport of inner Solar System material to the outer Solar System also. Together, these observations point to extensive material exchange between the inner and outer Solar System during its earliest stages, potentially associated with large-scale dynamical processes. The same collision may also have disrupted the CI parent body, and the associated heating may have generated the CI1T (previous CY1) chondrites (*33*, *79*).

### Volatile acquisition and depletion of the APB through impact

The angrite and CI chondrite parent bodies represent two compositional extremes in the Solar System, being among the most volatile-depleted (driest) and volatile-rich (wettest) bodies, in inner and outer Solar System, respectively. For example, angrites exhibit H_2_O contents and Rb/Sr ratios that are ∼10,000 and 3000 times lower, respectively, than those of CI chondrites (*15*, *80*–*82*). If ∼6% CI material had been added to the surface quenched angrite melt, a substantial increase in volatile element abundances, including water content and Rb/Sr ratios, would be expected in quenched angrites compared to deep-seated plutonic angrites. However, both matrix and mega-olivine phases in quenched angrites, as well as plutonic angrites, display comparable Rb/Sr ratios and H_2_O contents (*82*).

Petrologically, matrix and mega-olivine phases in quenched angrites may not be cogenetic (*25*, *83*). Their chemical and volatile similarities argue against a scenario in which erupted volcanic magma simply assimilated CI-rich material already present on the APB surface. Instead, it suggests that any volatile enrichment from the CI-like impactor was subsequently erased, likely due to volatile loss following the impact and mixing processes. This implies that the volatile inventory at the APB surface may have been reset, possibly as a consequence of the same impact that caused large-scale mantle melting on the APB (*27*). Thus, this impact should be high energy, causing a very high-temperature evaporation, e.g., up to 4000 K (*84*), which could have driven the evaporation of volatiles and volatile elements such as H_2_O, Rb, and K that were delivered by the CI material. The high-energy impact and high-temperature evaporation may also be the reasons for the extreme volatile-depletion signatures and heavy isotopic compositions of nonvolatile elements of angrites, e.g., Ni (*25*), Fe (*85*, *86*), and Si (*87*) and possibly Mg (*88*) with Tc_50%_ (50% condensation temperature) of 1363, 1338, 1314, and 1343 K, respectively (*89*), compared to chondrites and other differentiated bodies. The preservation of distinct ε^54^Cr and Δ^17^O signatures between surface and deep materials of APB argues against a fully disruptive giant impact and instead supports a high-energy but noncatastrophic planetesimal collision for the APB. This collision may be distinct from low-energy cratering events and Moon-forming–scale giant impacts that lead to large-scale re-accretion of planetary bodies. On the other hand, owing to the water-rich, sulfide-rich, and highly porous nature of CI chondrites (*90*), the CI parent body may have been disrupted during the impact. The associated heating could have metamorphosed portions of CI material, transforming them into CI1T chondrites, formerly CY1 chondrites (*33*, *79*). This interpretation is supported by their identical mass-independent O, Fe, Cr, and Ti isotope compositions (*33*, *79*, *91*) but contrasting H_2_O, sulfide, and volatile element contents (including Au), as well as distinct mass-dependent O isotope compositions (*33*, *63*, *79*, *92*).

 This high-energy impact scenario, together with subsequent rapid cooling on the APB, is also consistent with key petrographic observations. Vesicles, hollow shells, and augite-bearing druses observed in quenched angrites (e.g., D’Orbigny) indicate transient volatile or gas phases and open-system conditions during formation, as documented by detailed petrographic (*93*) and geochemical (*94*) studies. Regardless of genetic classification, these textures require short-lived volatile saturation followed by efficient volatile loss. In our model, the addition of volatile-rich CI-like material during a CI-angrite planetesimal collision, combined with impact-induced melting, would transiently drive the system to volatile saturation, followed by rapid decompression and high-temperature degassing under low-gravity conditions. This process naturally produces vesicles and hollow shells while preventing long-term volatile retention, reconciling textural evidence for transient fluids with the uniformly low present-day water contents of angrites.

This finding highlights an important, previously underappreciated mechanism for volatile depletion on differentiated bodies: Volatile-rich impactors can paradoxically enhance volatile loss if the collision generates sufficient thermal energy. For example, Earth (*95*–*98*) and Mars (*35*, *99*) are also believed to have received water and volatile elements from carbonaceous chondrite material. However, unlike the APB, they retained these volatiles after addition, due to their greater gravity. Such high-energy impacts could have played a key role in establishing the extremely dry nature of certain planetary materials, including angrites, and may help explain the volatile-depleted compositions of some terrestrial planets and asteroids. Our results underscore the need to consider impact-driven volatilization, rather than just initial accretion or internal differentiation, as a central process shaping the volatile budgets of early Solar System bodies.

Although volatiles may have been lost during this energetic event, the impact did not fully homogenize isotopic and elemental compositions. This is evident in the preserved heterogeneity in Δ^17^O and ε^54^Cr values between surface and deep materials, including the distinct ε^54^Cr values in the matrix of A 881371 (−0.15 ± 0.08) and A 12209 (0.27 ± 0.06) and the heterogeneous HSE contents among quenched angrites (*70*). Such isotopic and elemental heterogeneity likely reflects rapid cooling following the impact, which inhibited diffusive equilibration of O and Cr isotopes across the melt.

### Revisiting the bulk Mn-Cr isochrons of bulk angrites

Compared to the Mn-Cr isotope dating study by (*11*), our work incorporates a broader range of angrite samples, including dunite, olivine from dunite, mega-olivine from quenched angrites (the olivine-rich samples with lower ^55^Mn/^52^Cr ratio; Fig. 4 and Table 1), and matrix from quenched angrites. When all Mn-Cr data from this study and (*11*) are considered collectively, they define only an errorchron (MSWD = 24; Fig. 4A), with a slope of ∼0.272 and a *y* intercept of −0.019. This corresponds to an initial ^53^Mn/^55^Mn ratio of ∼3.08 × 10^−6^ and an absolute age of ∼4563.1 Ma, calculated using IsoplotR (*100*). The poor fit may reflect (i) contamination of the quenched angrite matrix by CI-like material or (ii) asynchronous formation of melt sources across angrite lithologies.

When only quenched angrites and their components are considered, the MSWD improves to 12, with a revised slope of ∼0.257 and an intercept of ∼0.079, corresponding to an initial ^53^Mn/^55^Mn ratio of ∼2.91 × 10^−6^ and an absolute age of ∼4562.8 Ma (Fig. 4B). This age discrepancy between quenched and plutonic angrites suggests their respective melt reservoirs formed at different times, consistent with the Mn-Cr and Pb-Pb ages of individual angrites [(*11*) and references therein]. However, this correlation for quenched angrites may represent a mixing line rather than a true isochron as the two mega-olivines exhibit low Mn/Cr ratios and high Cr contents, whereas the matrix samples have high Mn/Cr and low Cr contents. Now, the petrological relationship between mega-olivines and matrix in quenched angrites remains uncertain. Accordingly, we do not interpret the Mn-Cr trend for quenched angrites and their components as a meaningful age constraint.

In contrast, Fig. 4C shows that plutonic, intermediate, and dunite angrites, along with olivine from the dunite, define a well-resolved Mn-Cr isochron with a slope of 0.185 ± 0.028 and an intercept of −0.035 ± 0.030 (MSWD = 1.2, *N* = 6). This corresponds to a ^53^Mn/^55^Mn ratio that dates to 4561.0 ± 0.9 Ma, anchored to the D’Orbigny angrite. The final age uncertainty includes propagation of errors associated with the isochron slope, the half-life of ^53^Mn, the U-corrected Pb-Pb anchor age, and the initial ^53^Mn/^55^Mn ratio of the reference meteorite.

It is worth noting that the intermediate angrite NWA 10463 exhibits many textural features comparable to plutonic angrites and lacks the mega-olivines found in quenched angrites (*27*), suggesting its melt was likely derived from the same deep-seated reservoir as the plutonic and dunite angrites. Hence, this ^53^Mn-^53^Cr isochron plausibly records the timing of formation of the deep melt reservoir from which these angrites crystallized. The external isochron age is consistent with, or slightly older than, internal Mn-Cr or U-Pb isochron ages of plutonic angrites, ranging from 4560.74 ± 0.47 Ma (NWA 2999) to 4556.60 ± 0.26 Ma (Angra dos Reis) (*3*, *13*). These internal ages likely reflect progressive cooling and crystallization stages of the deep melt or different episodes of deep magmatism.

In contrast, the two mega-olivines from quenched angrites do not align with this Mn-Cr isochron, implying that they crystallized from a melt distinct from that which produced the plutonic and dunite angrites. The older internal isochron ages of quenched angrites, ∼4563 Ma (*3*, *12*, *13*, *21*, *22*, *101*), likely reflect the timing of near-surface volcanic activity on the APB, potentially triggered by impact events. This is consistent with the ε^54^Cr-based evidence for mixing with CI-like material discussed above.

Last, our results indicate that volcanic (quenched) angrites, including D’Orbigny, do not always represent closed isotopic systems, containing mega-olivines that may not be cogenetic to the matrix and may have experienced impact-related addition of chondritic material and loss of material. Such processes can disturb short-lived chronometer systematics, implying that these samples should be used with caution as absolute time anchors for short-lived chronometers, especially for those of siderophile elements such as Mn-Cr (*29*) and Fe-Ni (*102*) chronologies. In contrast, plutonic and dunitic angrites, as well as mantle-derived olivine separates, exhibit homogeneous ε^54^Cr compositions and define a well-resolved Mn-Cr isochron with low MSWD, consistent with closed-system behavior. These samples therefore appear to better preserve primary chronometric information and provide more robust temporal constraints on APB differentiation. More broadly, our findings highlight the importance of integrating isotopic tracers with chronometric systematics when selecting samples for early Solar System time anchors. For example, Zhu *et al.* (*2*) reported mineral-scale ε^54^Cr heterogeneities in the recently identified andesitic meteorite Erg Chech 002 (Table 1). Although Erg Chech 002 formed very early (4565 to 4566 Ma), within the lifetime of short-lived radionuclides such as ^26^Al, ^53^Mn, and ^60^Ni, and has been dated using multiple chronometers, such as U-Pb (*103*), Al-Mg (*1*), Mn-Cr (*2*), and Fe-Ni (*102*), the presence of isotopic heterogeneities and disequilibrium suggests that some chronometric systems may not record a single and well-defined age. Reevaluating established chronometers in this framework may refine absolute timelines of early Solar System events.

## MATERIALS AND METHODS

### Samples and Cr isotope analysis

Meteorite samples used in this study were provided by the National Institute of Polar Research (NIPR; Japan), the University of New Mexico Meteorite Museum (USA), and Z. Wang. The bulk angrites and matrix of quenched angrites were crushed into a fine powder using an agate mortar. Olivine grain samples are not crushed. Bulk plutonic and dunite angrite, MIL 07001, Erg Chech 002, Allende, and DTS-1 were processed in Parr bombs for ensuring full dissolution, e.g., chromites. For other samples, they were just used Savillex PFA vials for digestion, without Parr bombs. The procedure involved heating in ∼3 ml of concentrated HF and HNO_3_ (2:1) at 140°C for 2 days, which mainly dissolved silicates. After drying down, the samples are dissolved in ∼4 ml of concentrated HNO_3_ at 180°C for another 2 days to ensure complete digestion of carbon, organics, and tiny refractory phases such as chromite and spinel. After dissolution, all the samples were dried down and redissolved in 1 ml of 6 M HCl. After dissolution, no visible phases were observed in solutions.

For Cr purification and isotope analysis, 10% aliquots corresponding to 4–5 mg samples were taken up. We used a three-step column chemistry, mainly adapted from (*58*), (*104*), and (*5*), which are summarized in (*105*). The first column uses 1 ml of AG50W-X12 resin. Samples in 0.2 ml of 6 M HCl were diluted to 1 M HCl with 1 ml of Milli-Q water and then immediately loaded onto columns followed by 3.5 (1 + 1 + 1.5) ml of 1 M HCl to collect Cr. In this step, Cr was separated from most major elements, e.g., Fe, Mg, Ca, Mn, Ni, and V. To improve the chromatography yield for the first column, following the collection of the main ∼95% Cr elution, the remaining ∼5% Cr and sample matrix was collected using 3 (1 + 1 + 1) ml of 6 M HCl. This aliquot was then evaporated at 130°C to promote the formation of [CrCl_3_(H_2_O)_3_]^0^ and reloaded onto the first column, which was cleaned with 5 ml of 6 M HCl and reconditioned with 3 ml of 1 M HCl. In this way, Cr is well separated from V and Mg and a high Cr yield is maintained. The Cr from these two steps were then recombined and redissolved in 0.5 M HNO_3_ before loading on the third column. Before the third column, samples are dried down three times in 0.1 ml of concentrated HNO_3_ to convert to the nitrate form. Samples are then redissolved in 250 μl of 2 M HNO_3_ and then diluted with 730 μl of Milli-Q water to achieve ∼1 ml of ∼0.5 M HNO_3_. This solution was heated on a hotplate at 130°C for 2 hours to promote the formation of Cr^3+^ and equilibrium for the different species. After that, 20 μl of 30% H_2_O_2_ is added and the solution was kept at room temperature for 48 hours to maximize the promotion of Cr^3+^. The third column (homemade) contains 0.33 ml of Bio-Rad AG50W-X12 (200∼400 mesh) cation exchange resins. The resins were cleaned with 2 ml of 6 M HCl and 1 ml of Milli-Q water and then preconditioned with 1 ml of 0.5 M HNO_3_. Samples are loaded in 1 ml of 0.5 M HNO_3_, followed by 2.5 ml (1 + 1.5) 1 M HF to effectively remove residual Ti, Al, Fe, and V, and 6 ml (3 + 3) of 1 M HCl to remove the remaining Mg, K, Ca, and Na. Last, Cr was collected with 1.6 ml (0.4 + 0.4 + 0.4 + 0.4) of 6 M HCl. The yield of this column is higher than 95%. Compared to 3 ml of 6 M HCl for the final Cr collection with the same columns in a previous study (*31*), less acid is used in this step, minimizing the amount of organics from the resin and of potential Cr loss. The final Cr cut was dissolved in 0.75 ml of concentrated H_2_O_2_ (high purity) and 0.25 ml of concentrated HNO_3_ at 100°C overnight to destroy any organics that may affect the Cr ionization on a thermal ionization mass spectrometer. The total recovery of Cr for samples with different matrices (chondrites, basalts, and peridotites) from the three-column cation exchange chromatography procedure is higher than 92%, and the blank is less than 2 ng that is negligible relative to at least 10 μg of the Cr in the samples.

Cr isotope analyses were performed on a Thermo Fisher Scientific Triton Plus TIMS housed at the Institute of Science Tokyo. The final Cr cut was dried down and dissolved in 5 μl of 6 M HCl. Parafilm points were melted on the degassed W filaments to narrow down the size of sample loads. Then, 1 μl of the activator mixed by Al-doped Si gel and concentrated boric acid and 1 μl of the sample solution were loaded onto the center of filaments, between two parafilm points. Each sample was loaded onto two filaments to avoid potential “memory effects” of filaments. After drying down the solution on the filaments at current of 1.5 A, filaments were loaded onto a TIMS turret that was further loaded into TIMS. When the vacuum is better than 5 × 10^−7^ (without using liquid N_2_), we started the automatic measurements. The samples were measured by multistatic mode (*60*). Three lines were measured for each cycle, and the details can be found in (*60*). Each run includes 7 or 14 blocks, and one block contains 15 cycles with an integration time of 16 s. Gain calibration was done before each session (after changing the TIMS turret), and 30 s of baseline was measured before each block. The signal of ^52^Cr is automatically set as 10 to 13 V through all measurements. The ^53^Cr/^52^Cr and ^54^Cr/^52^Cr ratios were normalized to a ^50^Cr/^52^Cr ratio of 0.051859 (*106*) using an exponential law (*7*) and are expressed in the epsilon-notation (ε)

εCr=x(Cr/Cr52x)sample(Cr/Cr52x)NIST SRM 979−1×10,000 (1)

with *x* = 53 or 54.

The average 2SE uncertainties of ε^53^Cr and ε^54^Cr of this study are ∼0.04 and ∼0.08, and the external data uncertainty of the measurements is estimated as 0.06 and 0.12 for ε^53^Cr and ε^54^Cr, respectively, based on the 2SD variation of ε^53^Cr and ε^54^Cr of NIST SRM 979. We also tested our data accuracy by analyzing MIL 07001, DTS-1, Allende, and JP-1, and their data are consistent with the data produced from different labs and by different methods (*31*, *36*, *40*, *105*, *107*, *108*).

### Elemental content analysis

Major and trace element concentration measurements of the digested whole-rock aliquots were conducted on an Agilent 7700e ICP-MS at the Wuhan SampleSolution Analytical Technology Co. Ltd. (Wuhan, China). The relative standard deviation (RSD) uncertainty is estimated as 10%, based on multiple measurements of US Geological Survey (USGS) standards. We report the elemental data for two basaltic rocks BCR-2 and BHVO-2 measured during the same analytical sessions for data quality control. Similar methods were used in literature studies (*25*, *33*, *79*, *109*).

### Isotope mixing model by Monte Carlo calculation

Isotope ratios were modeled using a two-component binary mixing framework, where the isotopic composition of the mixture reflects concentration-weighted contributions from each endmember. Following the approach of Langmuir *et al.* (*73*), the ratio-element and ratio-ratio of isotopes are given by a hyperbola form

Ax+Bxy+Cy+D=0 (2)

where *x* and *y* are general variables along the abscissa and ordinate, respectively, and *A*, *B*, *C*, and *D* are coefficients of the general variables *x* and *y*; coefficients are described in detail in (*73*).

We applied a Monte Carlo forward-inversion method to model binary isotopic mixing. In our Monte Carlo framework, we generated a large ensemble of randomized mixing models by sampling the input parameters—including isotopic compositions (ε^54^Cr and Δ^17^O), elemental concentrations (Cr and O), and a mixing fraction of CI (*f*_CI_)—from normal distributions centered on their measured or assumed values, with SDs reflecting their reported 1σ uncertainties. For each realization, the isotopic ratio of the mixture was computed using the mixing model from Eq. 1. Simulated outputs were then compared to the measured isotopic values of the mixture. A solution was retained as valid only if the forward-calculated ε^54^Cr and/or Δ^17^O fell within ±2σ of the measured values and the inferred concentrations of the APB endmember and fraction of CI were positive and finite. This filtering produced a statistically robust subset of solutions that are internally consistent with all measured data and constraints. The mean of this filtered population was taken as the best estimate of the mixing parameters, and the 95% confidence intervals were derived from the 2.5th and 97.5th percentiles of the resulting distributions.

Oxygen contents were not measured directly by solution ICP-MS. We therefore estimated the oxygen contents of angrite samples stoichiometrically from the measured major and minor element abundances (table S1) by converting cation concentrations to simple oxides (e.g., FeO, MgO, CaO, Al_2_O_3_, Na_2_O, K_2_O, Cr_2_O_3_, MnO, TiO_2_, etc.), with SiO_2_ obtained by difference such that the sum of oxides equals 100 wt %. Total oxygen was then calculated from oxide stoichiometry, yielding an anhydrous stoichiometric oxygen content of 41.40 ± 0.21 wt % for the matrix of quenched angrites. Even under a conservative upper bound of residual water (≤1.1 wt % H_2_O) considering 6.29% CI contribution, the corresponding total oxygen content would increase only to ∼41.9 wt % (i.e., by ∼0.5 wt %); varying matrix O over this plausible range does not change the inferred CI fraction from the Monte Carlo model beyond the quoted 1σ uncertainty.

### Monte Carlo inversion for individual elements: Δ^17^O or ε^54^Cr with concentration

We first modeled binary mixing for individual isotopic systems, specifically, Δ^17^O with oxygen concentration (fig. S3) and ε^54^Cr with chromium concentration (fig. S4). For each case, 10^5^ realizations of the *f*_CI_ were drawn from a truncated normal distribution (constrained to [0,1]), with isotopic compositions and concentrations sampled from Gaussian distributions based on known means and 1σ uncertainties. Isotopic values (Δ or ε) were converted to ratios using natural abundance scaling. Using mass balance equations and known endmember CI values, the unknown concentration of endmember APB was computed. The resulting *f*_CI_, *C*_APB_, and calculated isotope values were visualized with two-dimensional (2D) scatterplots and confidence intervals.

The Δ^17^O-O content inversion (Fig. 3A) yields a fraction of CI (*f*_CI_) of 9.54 ± 3.61% and an inferred APB oxygen content of ∼40.8 ± 0.7 wt %, consistent with the angrite whole-rock compositions [40.0 wt %; (*22*)]. The ε^54^Cr-Cr inversion (Fig. 3B) yields a *f*_CI_ of 5.01 ± 2.49% and an APB Cr content of 451 ± 95 μg/g. The two single-system estimates overlap within 2σ uncertainties, consistent with a common CI-APB mixing scenario.

### Joint Monte Carlo inversion of ε^54^Cr and Δ^17^O

To improve constraint resolution, we developed a combined Monte Carlo model that simultaneously solves for binary mixing of ε^54^Cr and Δ^17^O with associated elemental concentrations (Fig. 3C and fig. S5). In this approach, a shared vector of *f*_CI_ values generated using a truncated Gaussian distribution, centered on prior estimates and constrained to [0,1], was used to jointly sample isotopic and concentration values for both Cr and O endmembers. For each Monte Carlo realization (here we increase N to 10^6^), forward-modeled ε^54^Cr and Δ^17^O values were simultaneously computed from concentration-weighted isotope ratios.

This dual-filtering approach narrows the solution space, yielding a set of internally consistent and geochemically plausible models. The valid subset of simulations represents the region in parameter space where both isotopic systems are consistent with the measured mixture, given all uncertainties. Results were evaluated through marginal distributions (mean, SD, and 95% confidence interval) and 2D scatterplots, including a combined ε^54^Cr versus Δ^17^O plot with confidence boxes, kernel density contours, and a covariance ellipse. This joint inversion yields a CI fraction of 6.29 ± 1.97%, which we adopt as our preferred estimate.

In the main analysis, the joint inversion uses the mean of the two matrix separates as the matrix constraint. As a robustness test, we repeated the joint inversion treating the two matrix separates of A 881371 and A 12209 as individual constraints (fig. S5). In this implementation, ε^54^Cr and elemental abundances (Cr and stoichiometric O) are applied separately to each matrix separate, whereas Δ^17^O for the matrix is taken from published high-precision measurements. The resulting CI fraction is indistinguishable from the mean-matrix approach, yielding *f*_CI_ = 6.13 ± 0.82%.

Although our joint Monte Carlo model successfully reproduces the measured ε^54^Cr and Δ^17^O values within 2σ uncertainty, we note that the mean of the valid simulation outputs does not lie exactly on the measured mixture point (Fig. 3C and fig. S5), due to possible asymmetric propagation of uncertainties, the nonlinear relationship between isotopic ratios and Δ/ε values, and the filtering criteria imposed during model selection. This slight offset is expected in forward Monte Carlo models and reflects the valid distribution shaped by all sources of uncertainty. The measured point lies well within the 95% confidence region, confirming the robustness and consistency of the model.
