# Supplementary material for: When wet meets dry: An Ivuna-like impactor triggered volatile loss on the angrite parent body
Source: Sci Adv. 2026 May 29;12(22):eaeb1432. doi: 10.1126/sciadv.aeb1432 (PMC13220875; doi:10.1126/sciadv.aeb1432)
Supplement: Supplementary file 1 — Figs. S1 to S5 Table S1 [file sciadv.aeb1432_sm.pdf]

Science Advances
AAAS

Supplementary Materials for

When wet meets dry: An Ivuna-like impactor triggered volatile loss on the angrite parent body

Ke Zhu *et al.*

Corresponding author: Ke Zhu, zhuke@cug.edu.cn, ke.zhu@bristol.ac.uk

Sci. Adv. **12**, eaeb1432 (2026)
DOI: 10.1126/sciadv.aeb1432

This PDF file includes:

Figs. S1 to S5
Table S1

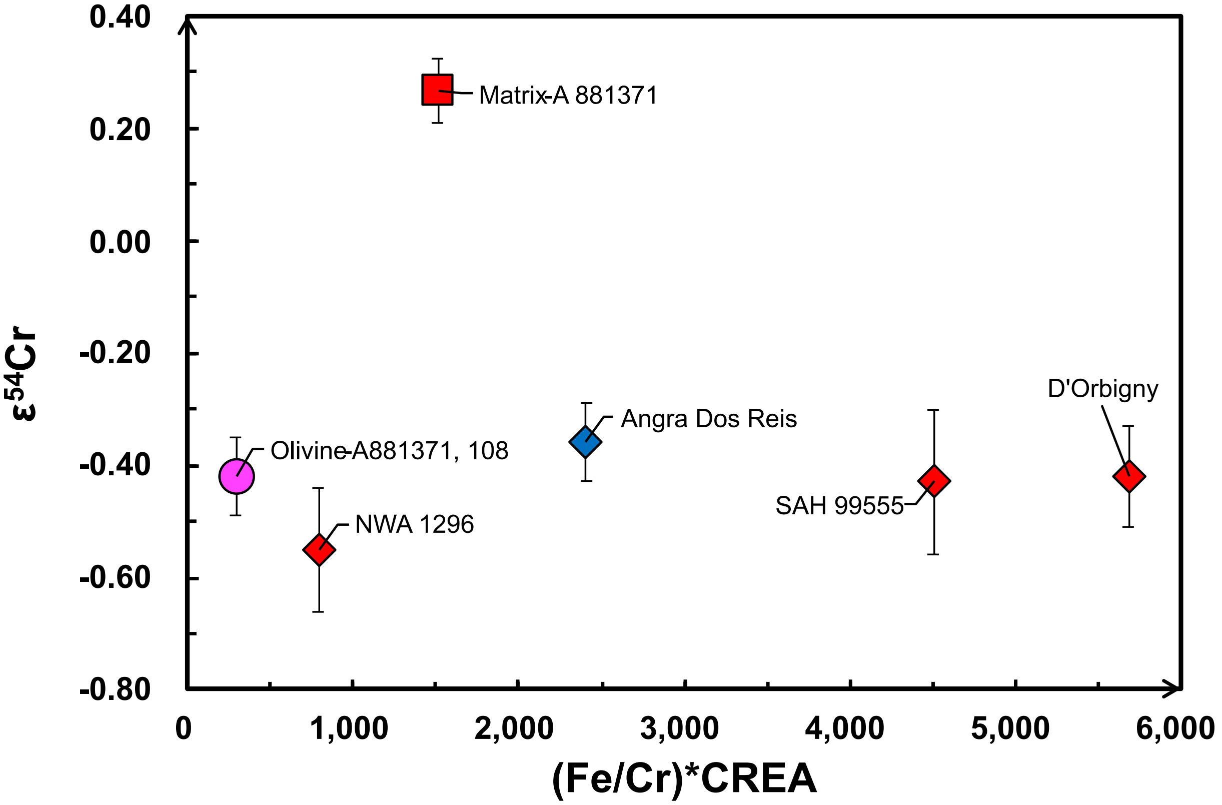

Fig. S1. Plot of Fe/Cr ratios × cosmic-ray exposure ages and ε^{54}Cr values, with data sources (*11, 56*). Lack of correlation between the two indices means spallation cosmogenic effect on Cr isotopes in angrites is limited. There is no literature CREA data of A 12209.

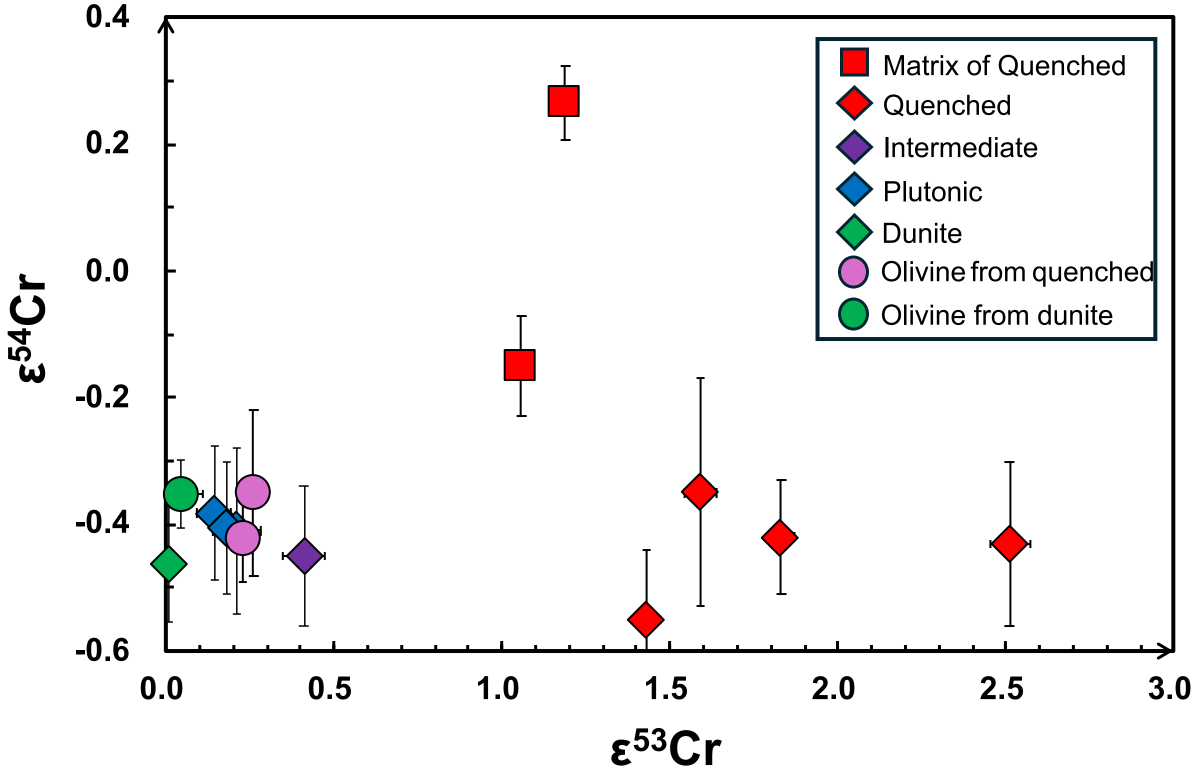

Fig. S2. Plot of ε^{53}Cr and ε^{54}Cr values for angrites, with data sources (*11*). The absence of correlation between the two indices means the two anomalously high ε^{54}Cr values (matrix of A 881371 and A 12209) should not be caused by analytical artefacts, such as non-kinetic Cr isotope fractionations in low yield of column chemistry (*36*) or TIMS evaporation (*31, 59, 60*).

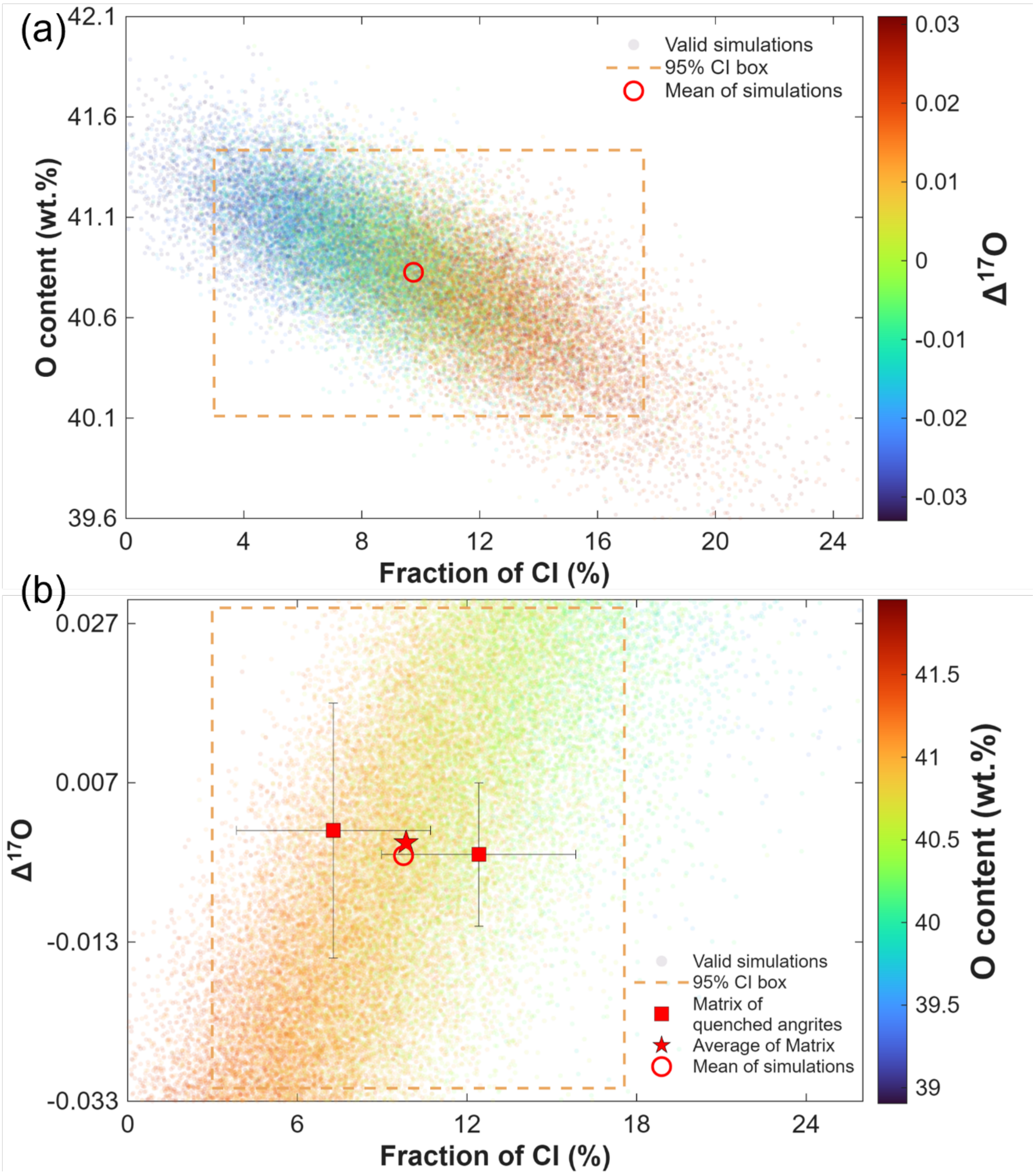

Fig. S3. Single-system Monte Carlo inversion of Δ^{17}O-O constraints for CI-APB mixing. (a) Valid simulation showing the relationship between CI mass fraction (f_{CI}) and the inferred APB oxygen content (wt.%); points are colored by the forward-modeled Δ^{17}O of the mixture. (b) f_{CI} versus the mixing Δ^{17}O, colored by modeled APB O content. In panel (b), red squares denote the two volcanic angrite matrix (A-881371 and A-12209) with uncertainties (Δ^{17}O from Rider-Stokes et al (*27*)., 2023); the red star marks the mean matrix composition. The dashed orange box encloses the central 95% credible region of accepted solutions, and the open red circle indicates the mean of the valid simulations. The inversion yields f_{CI} = 9.54 ± 3.61% and an inferred APB oxygen content of ~40.8 ± 0.7 wt.%.

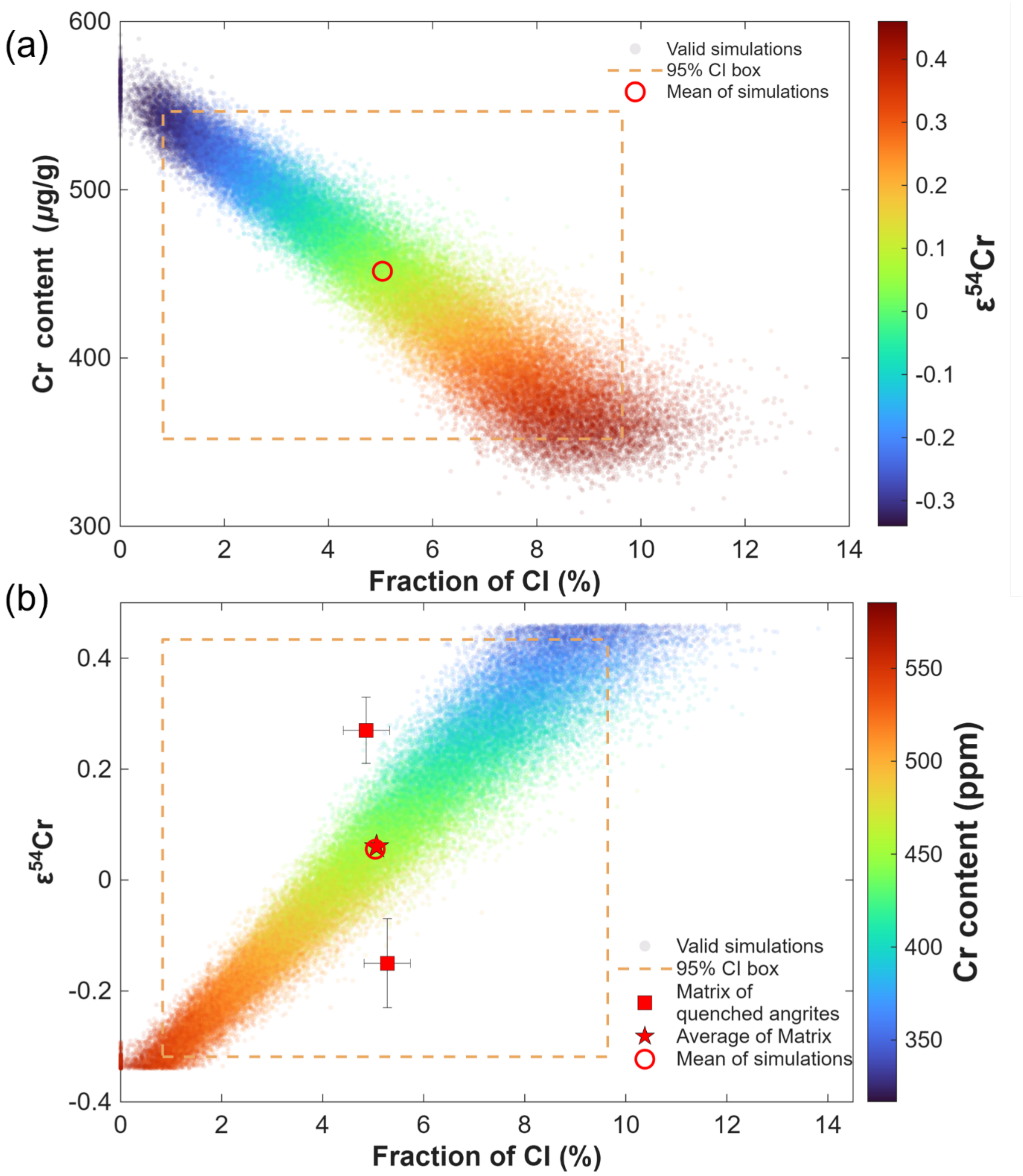

Fig. S4. Single-system Monte Carlo inversion of ε^{54}Cr-Cr constraints for CI-APB mixing. (a) f_{CI} versus inferred APB Cr concentration, colored by the modeled mixingε^{54}Cr. (b) f_{CI} versus mixing ε^{54}Cr, colored by modeled APB Cr concentration. Symbols and uncertainty conventions are as in Fig. S3. The inversion yields f_{CI} = 5.01 ± 2.49% (2σ) and an inferred APB Cr concentration of 451 ± 95 ppm.

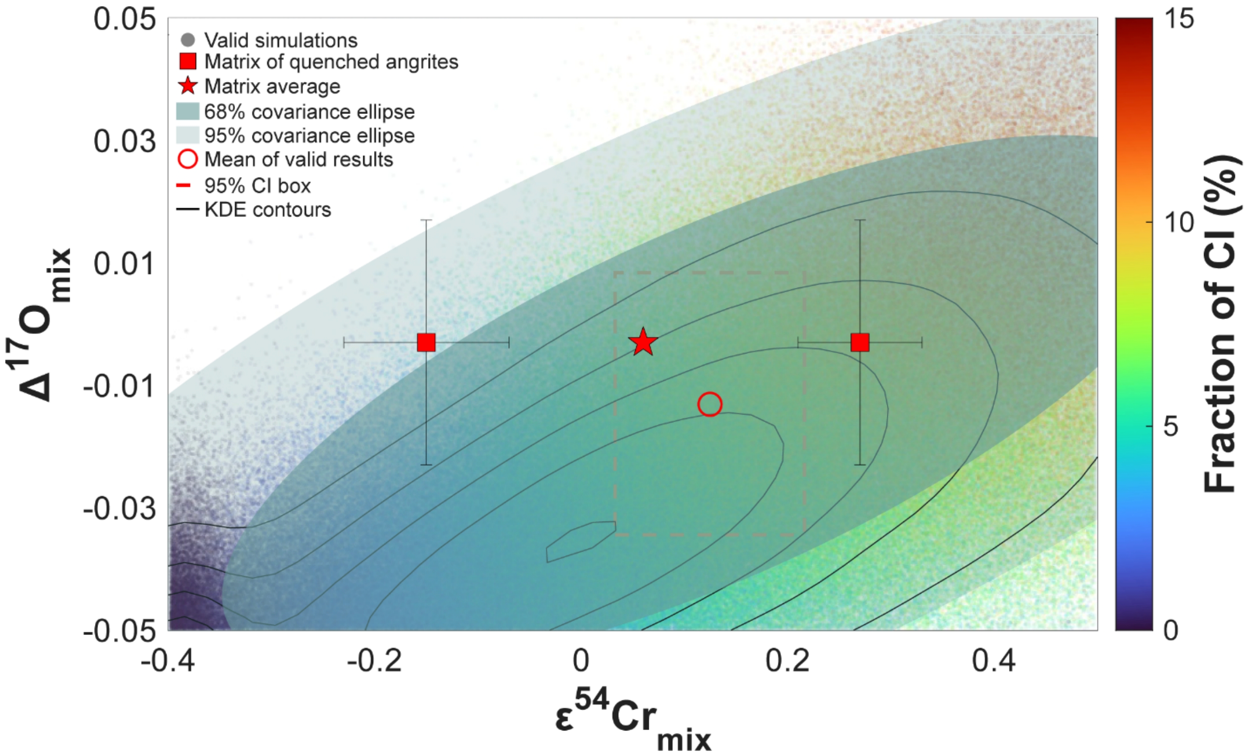

Figure S5. Joint Monte Carlo inversion of $\Delta^{17}O$ versus $\varepsilon^{54}Cr$. The "mix" means the matrix of quenched angrites were mixed by APB and CI material. The inversion was performed treating the two volcanic angrite matrix separates (A 881371 and A 12209; red squares with ± 2σ uncertainties) as independent matrix constraints rather than a matrix mean. Valid Monte Carlo realizations that simultaneously reproduce the matrix $\Delta^{17}O$ and $\varepsilon^{54}Cr$ constraints within ± 2σ are shown as colored points, where color denotes the inferred CI fraction. The inversion yields $f_{CI} = 6.13 \pm 0.82$ % (2σ). The open red circle indicates the mean of the valid simulations, and the red star shows the mean value of the matrix. Black contours show kernel density estimation (KDE) of valid-simulation density; the dashed orange rectangle encloses the central 95% credible region, and the shaded ellipses indicate the 68% and 95% covariance structure of the valid-simulation distribution.

Table S1 Chemical compositions of angrite samples

Sample Name	Bulk-NWA 8535	Olivine-NWA 8535	Matrix A 881371	Olivine-A881371	Matrix A 12209	Olivine-A12209	NWA 14758	NWA 14758	Rafsa 005	EC 002	BCR-2	BHVO-2
Sample type	Dunite	Olivine	Matrix	Olivine	Matrix	Olivine	Plutonic	Plutonic	Plutonic	Ungrouped Achondrite	Terrestrial basalt	Terrestrial basalt
Li	0.57	0.47	4.00	0.64	4.09	0.65	3.15	3.17	3.56	2.50	10.00	4.41
Be	ND	ND	0.36	0.01	0.35	ND	0.03	0.05	0.06	0.13	2.10	1.12
Na	38.5	44.0	172.5	32.3	165.1	25.7	64.7	65.7	84.9	32439	25403	17331
Mg	234180	259006	53386	276091	48654	285046	103332	103473	113753	39380	23367	46121
Al	5583	2430	54481	1150	53699	970	17179	17469	32399	48323	76415	74897
P	46.4	62.2	622.6	48.2	700.8	81.7	90.2	97.3	104.6	533.3	1693.2	1254.3
K	ND	ND	ND	ND	ND	ND	ND	ND	ND	3832	16022	4741
Ca	2864	2234	87271	2551	92422	2466	46607	46939	52085	53604	54307	84095
Sc	5.6	4.3	35.9	5.0	38.2	4.7	18.2	18.5	15.8	29.5	36.8	34.3
Ti	152	117	4398	93	4828	121	1443	1373	2207	2526	14514	17425
V	79.6	39.1	104.3	29.0	108.7	25.2	72.5	73.0	60.4	82.4	444.5	334.1
Cr	5673	3060	557	1544	566	1560	2208	2266	1732	3526	16.2	290
Mn	1212	1125	1808	1019	1910	989	1789	1832	1929	3387	1672	1391
Fe	108413	99615	167037	88460	172531	84746	250389	254017	234009	80959	104812	91917
Co	62.40	60.56	39.81	48.66	41.00	50.06	344.51	344.75	226.30	4.35	39.64	46.70
Ni	197.1	187.1	89.2	80.0	108.4	84.6	5057.8	5124.0	3225.4	6.4	13.4	119.5
Cu	1.00	0.74	4.41	0.95	4.29	0.40	7.28	7.62	6.15	2.97	21.62	135.22
Zn	4.48	8.96	6.93	8.94	5.24	6.55	8.33	8.14	9.61	3.95	148.02	115.03
Ga	0.11	0.09	0.57	0.05	0.52	0.04	0.67	0.66	0.67	3.42	24.47	22.91
Rb	0.037	0.052	0.115	0.045	0.062	0.030	0.083	0.085	0.297	12.212	52.084	9.363
Sr	0.660	0.626	87.37	0.456	93.74	0.206	33.84	34.69	66.38	51.93	358	409
Y	0.312	0.215	16.160	0.212	18.114	0.188	6.415	6.674	6.371	9.399	38.144	27.011
Zr	0.65	2.55	45.04	0.82	49.67	0.25	16.81	17.05	19.07	25.83	198.20	177.22
Nb	0.099	0.585	2.940	0.305	3.273	0.053	0.092	0.014	0.236	1.955	13.419	19.363
Sn	0.189	0.409	0.657	0.250	1.391	0.492	0.155	0.179	0.205	0.271	2.406	2.018
Cs	0.004	0.007	0.009	0.005	0.009	0.001	0.008	0.006	0.016	0.827	1.258	0.104
Ba	0.41	0.86	24.71	0.63	26.80	0.30	179.62	182.65	176.78	36.27	730.12	137.74
La	0.034	0.027	2.701	0.030	3.052	0.007	0.746	0.758	0.605	1.581	27.700	16.311
Ce	0.079	0.069	7.124	0.060	7.819	0.017	1.898	1.886	1.658	4.123	58.496	40.602
Pr	0.013	0.011	1.126	0.009	1.245	0.002	0.314	0.327	0.293	0.648	7.439	5.673
Nd	0.060	0.038	5.754	0.049	6.287	0.007	1.718	1.751	1.568	3.237	30.365	24.665
Sm	0.018	0.016	1.817	0.012	2.031	0.003	0.613	0.669	0.557	1.060	7.864	6.828
Eu	0.010	0.007	0.701	0.005	0.749	ND	0.233	0.246	0.307	0.365	2.191	2.188
Gd	0.031	0.018	2.394	0.017	2.636	0.029	0.971	0.945	0.880	1.417	7.078	6.282
Tb	0.006	0.006	0.431	0.003	0.488	0.002	0.157	0.167	0.162	0.259	1.169	1.017
Dy	0.051	0.036	2.960	0.024	3.302	0.023	1.123	1.115	1.093	1.725	6.912	5.764
Ho	0.012	0.007	0.639	0.010	0.739	0.006	0.228	0.238	0.230	0.373	1.420	1.030
Er	0.034	0.033	1.865	0.029	2.035	0.026	0.722	0.693	0.642	1.094	3.958	2.658
Tm	0.008	0.009	0.274	0.011	0.294	0.005	0.117	0.113	0.111	0.168	0.559	0.367
Yb	0.056	0.044	1.721	0.053	1.951	0.054	0.706	0.735	0.747	1.058	3.784	2.172
Lu	0.013	0.010	0.252	0.012	0.304	0.007	0.118	0.121	0.124	0.160	0.565	0.286
Hf	0.025	0.092	1.258	0.031	1.410	0.014	0.516	0.515	0.604	0.760	5.445	4.857
Ta	0.005	0.014	0.157	0.011	0.182	0.005	0.009	0.004	0.026	0.094	0.820	1.220
Tl	0.005	0.010	0.006	0.008	0.004	0.008	0.022	0.020	0.015	0.016	0.312	0.078
Pb	0.047	0.155	0.196	0.079	0.187	0.111	1.434	1.428	3.287	1.902	11.140	1.479
Th	0.008	0.008	0.349	0.008	0.415	0.002	0.034	0.039	0.049	0.215	6.416	1.297
U	0.003	0.006	0.101	0.005	0.118	0.001	0.108	0.105	0.055	0.091	1.736	0.432

Notes: The units for all elemental contents are μg/g (ppm). ND means not determined. Some of the elemental data have been reported in (*25*).
